# Primary peritoneal high-grade serous carcinoma in a man: A case report

**DOI:** 10.1016/j.amsu.2022.103605

**Published:** 2022-04-12

**Authors:** Abdelali Guellil, Rachid Jabi, Mohamed Yassine Mabrouk, Laila Bouzayan, Abdelali Merhoum, Gérald Del Gallo, Claire Godart, Mohammed Bouziane

**Affiliations:** a Department of General Surgery, Mohammed VI University Hospital, Oujda, Morocco; b Faculty of Medicine and Pharmacy, Laboratory of Anatomy, Microsurgery and Surgery Experimental and Medical Simulation (LAMCESM), Mohammed Ist University, Oujda, Morocco; c Department of Uro-digestive and Bariatric Surgery, Charles Nicolle University Hospital, Dieppe, France

**Keywords:** High-grade primary peritoneal serous carcinoma, Male, Diagnosis, Histology

## Abstract

**Introduction and importance:**

Primary peritoneal serous carcinomas (PPSC) are exceedingly rare in male patients. Only a few cases were reported in the medical literature, it's diagnosis is difficult before surgery.

**Case presentation:**

In this article, we describe the case of a patient who presented a high-grade primary peritoneal carcinoma, the diagnosis was suspected radiologically following an abdominopelvic computed tomography (CT).the patient underwent exploratory laparoscopic surgery with biopsy of several peritoneal nodules. Pathologic analysis of specimen confirmed the diagnosis of Primary peritoneal serous carcinomas. The patient died one month after his diagnosis while undergoing chemotherapy and palliative care.

**Clinical discussion:**

PPSC is an inoperable malignancy, histology staining confirms the diagnosis, the chemotherapy and palliative care are the only offered treatment. The evolution of the disease is very dark with a poor prognosis.

**Conclusion:**

We highlight the important of testicular examination to predict apparition of PPSC in the future.

## Highlights

• Primary carcinoma of the peritoneum of high grade is a rare entity.
• Surgical exploration with peritoneal biopsy helps in the diagnosis.
• Immuno-histochemical analysis and molecular analysis are the gold standard of the diagnosis of this rare entity.
• Early chemotherapy is the first-line treatment.
• The median survival time of primary peritoneal carcinoma is 1–3 months, which means a poor prognosis.

## 1 Introduction

Primary peritoneal serous carcinoma (PPSC) is a clinicopathological entity, which was described exclusively in women. The male: female ratio ranges from 0.0018 to 0.0045 [1].

The international literature lists 217 cases reported between 1974 and 2006 (215 in women and 2 for men, age between 33 and 70 years) [2]. This type of cancer arises from the peritoneal epithelium and is similar to serous ovarian carcinoma. The hypothesis that serous carcinomas arise from Müllerian tissue fits well with the observation of their rarity in men. A diagnosis of PPSC is typically made based on the Gynecology Oncology Group criteria [3]. However, a correct differential diagnosis of PPSC is difficult preoperatively.

In this report, we will describe a case of a male patient suffering from a primary serous carcinoma of the peritoneum on the clinical, radiological and anatomopathological level.

This work has been reported following SCARE 2020 guidelines [4].

## 2 Case report

**Fig. 1:**
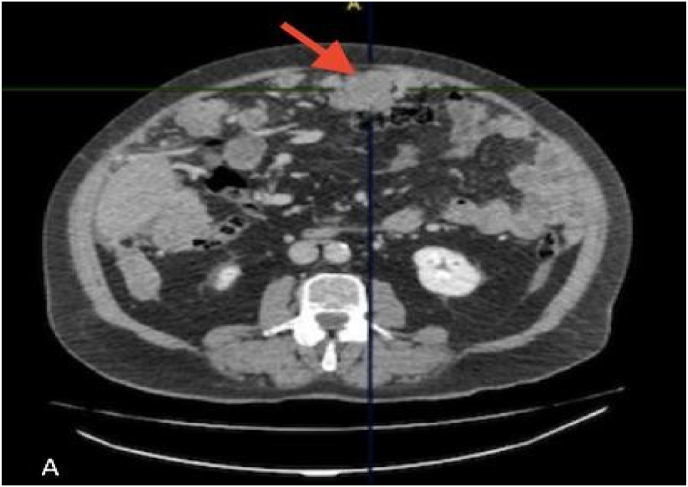
A: Axial section of primary serous carcinoma of the peritoneum in a 77-year-old man showing the presence of a large dense nodular lesion with lobulated contours measuring 6 cm.

**Fig. 2:**
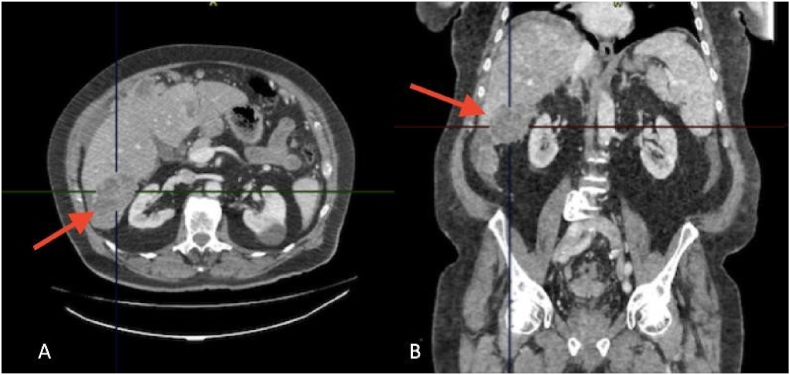
Visualization of multiple focal intrahepatic lesions of secondary appearance. The two target lesions are located in segment VIII (coronal section B), measuring 66 mm and 50 mm in maximum diameter. Another lesion is found in segment VII(axial section A), 4th lesion in segment III.

We present a case of a 77-year-old patient, active smoker, hypertensive under therapy calcium channel blocker 10 mg, valsartan 160 mg and hydrochlorothiazide 12.5, with a pace maker in 2018, already operated with the placement of a total knee prosthesis for gonarthrosis at an advanced stage, vaccination against COVID received, and he has no declared allergic history. Seen in the emergency room of Dieppe hospital for abdominal pain and hepatic colic, with palpation of a mass in the right hypochondrium. Testicular examination revealed no abnormality and the rectal exam was negative with no trace of blood. The patient was referred to a CT scan which revealed multiple focal intrahepatic lesions of secondary appearance, with peritoneal carcinosis and ascites of low abundance (Fig. 1, A) (Fig. 2 A and B).

**Fig. 3:**
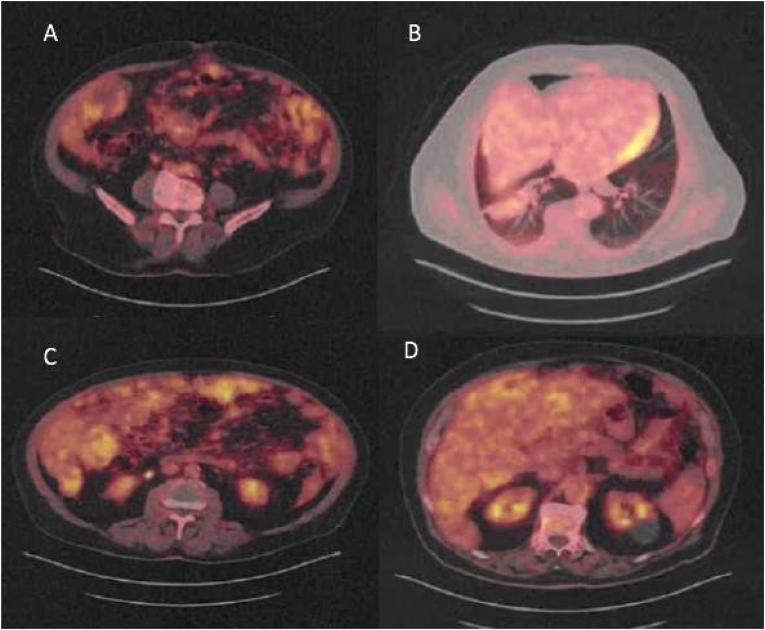
Positron emission tomography-computed tomography (PET/CT) finding. The nodular mass lesion shows intense FDG uptake in segment VII (B, D). Moderate to intense FDG uptake on large peripheral peritoneal structures (A, C).

Positron emission tomography with 18F-FDG showed heterogeneity of hepatic fixation with intense hypermetabolism around hypodense lesions, notably the tip of segment VI (SUVmax 6.5), the left liver (SUVmax = 5.6), with moderate to intense hypermetabolism around voluminous peritoneal tissue structures at the perihepatic, subparietal (SUVmax 6.3), right and left parietal gutters, intramesenteric and pelvic levels (SUVmax 6.2). In parallel, an echo-guided liver biopsy was performed without any particularity (Fig. 3).

**Fig. 4:**
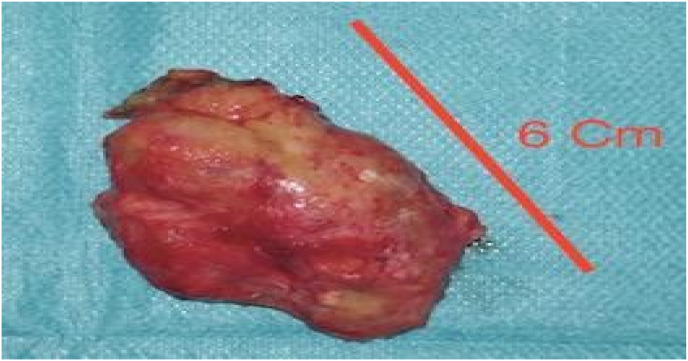
Exeresis part of our exploratory laparoscopy.

In the absence of a diagnosis of a primary tumor, the general surgery team at the Dieppe hospital center, in consultation with the patient, decided to perform an exploratory laparoscopy, which revealed numerous nodules ranging from a few millimeters to 6 cm in the parietal peritoneum (Fig. 4), in the hypochondria, but also the pelvis, and above all, a nodular greater omentum. Note that the appearance is pinkish and not whitish as is usual. It should be noted that the surgical procedure went well, and the postoperative care in the surgical department was uneventful.

**Fig. 5:**
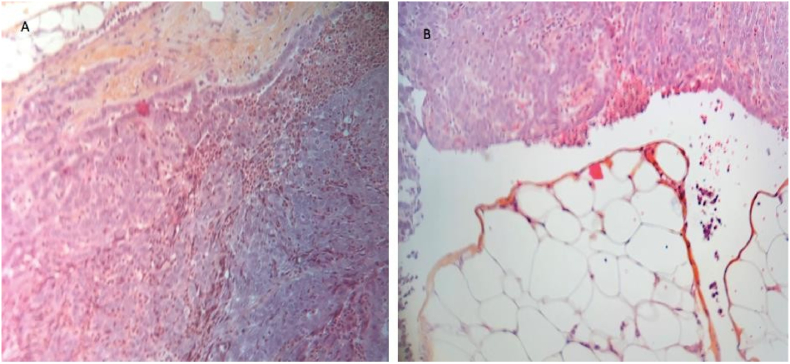
Typical histological features of high-grade serous carcinoma. A. Nests of tumor cells separated from the surrounding stroma by a retraction space and associated with numerous psammoma bodies (hematoxylin-eosin, original magnification × 200). B. The tumor cells show high-grade nuclear atypia without conspicuous mitotic activity (hematoxylin-eosin, original magnification × 200).

Histological analysis of our specimen (6 cm) showed a tubulo-papillary tumor proliferation made of atypical columnar cells accompanied by mitoses and large patches of necrosis, the immunhistochemical study in kerosene was positive to EMA, Keratin 7, Calretinin, BER EP4, HBME, CD15, P53 and negative to desmin Keratin 20, CD X2, TTF-1. These morphological and immunophenotypic results suggested a primary serous tumor of the peritoneum of high grade in a man, so we deepened our investigations in clinical and paraclinical examination of the testicle and paratesticular but without contributory results (Fig. 5).

The primary lesion was the omentum; the patient was diagnosed with PPSC, and chemotherapy was performed as first-line treatment with TAXOL and CARBOPLATAIN. Unfortunately, the patient died one month after his diagnosis while undergoing chemotherapy and palliative care. No autopsy was performed.

## 3 Discussion

Primary serous peritoneal carcinoma is a rare malignancy with an extremely low prevalence in men (1).It is a papillary tumor with psammatic bodies resembling ovarian carcinoma originating from the pelvic peritoneum [5]. It is classified in the same way as its ovarian counterpart into low and high grade serous carcinoma [6,7]. In the literature there are only 5 cases of primary serous carcinoma of the peritoneum in men [7].

Common clinical symptoms of PPSC are abdominal distension, abdominal pain, and discomfort. These symptoms are similar to those of peritoneal carcinomatosis. Oh Jisoo et al. [8] found that PET/CT is useful to evaluate the origin of the tumor, its extent and distant metastasis which is the case for our case, Therefore, it is difficult to diagnose PPSC before surgery which helps to visualize well the appearance of the tumor which is different from peritoneal carcinomatosis and make accurate biopsies on the peritoneal lesions contributing to have correct and rapid anatomopathological results.

Our PPSC case was immunihistochemically studied and positive for P53 and EMA, PAX8 staining was not done. There are only 2 patients (the patient of Shmueli et al. [9] and the patient of Shah et al. [10]with recently reported high grade immunophenotypic status. EMA and CK 7 is diffusely positive in all 3 cases. In addition, two of the three cases had high-grade tumor morphology and P53 testing was not performed, which may call into question the validity of these few cases. The reported immunphenotypes of these tumours, as well as that of our tumor, are summarized in Table 1.

**Table 1:** Different reports studing immunophenotype of peritoneal carcinoma.

Year reported	Age	histology	Positive stains (IHC)	negative stains (IHC)	Survival status
2021 Current patient	77	High-grade	CK7 EMA Calrétinine BER EP4 HBME CD15 P53	CK20 Desmine CD X2 TTF-1	Deceased (3 months)
2001 (Shmueli et al.)	53	High-grade	CK7 CK20 HMWK LMWK EMA CD15	ER/PR CEA Vimentin	Deceased (2 months)
1998 (Shah et al.)	74	High-grade	CK7 CK20 EMA BerEP4 PR (rare cells) CAM5.2	B72.3 Vimentin Thyroglobulin Chromogranin Synaptophysin	Deceased (1 months)

tbbreviations: (8), carcinoembryonic antigen; CK, cytokeratin; EMA, epithelial membrane antigen; ER, estrogen receptor; HMWK, high molecular weight cytokeratin; IHC, immunohistochemistry; LMWK, low molecular weight cytokeratin; PR, progesterone receptor; TTF-1, thyroid transcription factor-1.

Given the rarity of extragonadal serous carcinomas in humans, the above findings are not sufficient, in our opinion, to identify our 3 cases as Müller-type serous carcinomas. During foetal development of the male embryo, the Müllerian ducts develop but disappear within 9–12 weeks under the influence of Müller inhibitory factor (MIF) [9]. The foetal testes secrete this factor, which is responsible for the inhibition of Müllerian duct development. This temporary presence of Müllerian ducts in the male embryo may explain the rare occurrence of lesions resembling papillary serous ovarian cancer in the adult female [9]. In our patient's case, testicular examination and subsequent testicular ultrasound did not definitively demonstrate a mass lesion. However, since no autopsy was performed, we cannot completely rule out the possibility of a small occult gonadal primary tumor. Of note, the patient had no history of solid organ transplantation from a female donor that could have served as a possible source of tumor.

Although it is difficult to generalize, because some published survival data were from small studies, the median survival time for patients with PPSC is 11–17 months [11]. in contrast to the other 3 cases, which are 1–3 months.

The interest in early surgical peritoneal biopsy to confirm the primary peritoneal origin before reaching the stage of peritoneal carcinosis is stressed.

## 4 Conclusion

Because of its rarity, a detailed clinical and especially urological examination, molecular analysis, and multicenter immunohistochemical studies as well as autopsy are necessary to better understand the pathogenesis and to help diagnose and identify an effective therapeutic approach for this rare entity.

## Ethical approval

No ethical approval necessary.

## Sources of funding

The author(s) received no financial support for the research, authorship and/or publication of this article.

## Author contribution

**Dr Guellil Abdelali:** Have written the article, have consulted the patient, prescribed all of the tests and prepared the patient for surgery and participated in the surgery. **Dr Mabrouk Mohamed Yassine:** Have helped writing the article, data collection. **Dr Merhoum Abdelali:** Interpretation of pathological data. **Dr Bouzayan Laila:** Data collection. **Dr Del Gallo Gérald:** correction of the article, and scientific research. **Dr Claire Godart:** Data collection. **Pr Jabi Rachid:** supervised the writing of manuscript. **Pr Bouziane Mohammed** (oncology surgery professor): have supervised the writing of the paper, and has been the leader surgeon of the case.

## Research registration number

Our paper is a case report; no registration was done for it.

## Guarantor

Guellil abdelali

## Consent

Written informed consent was obtained from the patient for publication of this case report and accompanying images. A copy of the written consent is available for review by the Editor-in-Chief of this journal on request.

## Provenance and peer review

Not commissioned, externally peer-reviewed.

## Declaration of competing interest

The authors declared no potential conflicts of interests with respect to research, authorship and/or publication of the article.
